# Modulation of transmembrane domain architecture by the intracellular C-terminal domain in yeast glucose transporter–receptor divergence

**DOI:** 10.3389/fmolb.2025.1702400

**Published:** 2025-12-12

**Authors:** Jeong-Ho Kim

**Affiliations:** Division of Health and Natural Sciences, College of Arts and Sciences, Columbia International University, Columbia, SC, United States

**Keywords:** glucose, receptors, transporters, C-terminal domain, glucose-binding

## Introduction

The yeast glucose-sensing receptors Rgt2 and Snf3 offer one of the most fascinating examples of evolutionary innovation within the Major Facilitator Superfamily (MFS) (Ozcan et al., 1998; Ozcan et al., 1996). These paralogous plasma membrane proteins appear to have repurposed their transporter-like architecture for a completely different role—sensing rather than transport (Johnston and Kim, 2005; Kim et al., 2013; Ozcan and Johnston, 1999). Yet, despite decades of work, we still do not fully understand how a transporter becomes a receptor. The persistence of key substrate-binding residues only deepens this mystery—suggesting that the distinction between “transporter” and “receptor” may be far more fluid than we once assumed.

Emerging structural and mechanistic studies provide clues. In normal transporters, glucose binding drives a conformational transition known as the “alternating access” mechanism—opening outward, then inward, to shuttle sugar across the membrane (Custodio et al., 2021; Drew et al., 2021; Yan, 2017).

In Rgt2 and Snf3, however, this conformational choreography appears to be arrested: upon glucose binding, the receptor adopts a signaling-competent state that prevents completion of the transport cycle, representing an adaptation optimized for detection rather than substrate translocation (Scharff-Poulsen et al., 2018). This reflects its evolutionary shift from transporter to glucose receptor, prioritizing signaling over transport.

What truly distinguishes the yeast glucose sensing receptors (YGSRs) is their unusually long cytoplasmic C-terminal tails. Often overlooked in structural models, these domains are critical signaling elements. Evidence indicates that they not only recruit downstream regulators such as Mth1 and Std1 but may also allosterically modulate the transmembrane core itself (Kim et al., 2024; Roy et al., 2015; Schmidt et al., 1999). This coupling between the intracellular and transmembrane domains may be key to understanding how a single structural scaffold can simultaneously mediate both transport and signaling functions. The interplay between intracellular domain architecture and the transmembrane core is increasingly recognized as a major determinant of MFS transporter functional modality(Lee et al., 2016; Mikros and Diallinas, 2019; Muraoka et al., 1995).

Recent studies using Hxt1-based chimeras fused to the Rgt2 or Snf3 tails suggest a compelling principle: the cytoplasmic tail can reshape the behavior of the transporter core, highlighting that domain–domain communication, rather than the transmembrane elements alone, is likely the key determinant of whether an MFS protein functions as a transporter or a receptor. This insight frames the perspective explored in this article.

## Yeast and human glucose transporters share conserved transport mechanisms

In human GLUT1, residues Q161 (TM5), N288 (TM7), N317 (TM8), and W412 (TM10) form the core of the substrate-binding pocket (Deng et al., 2014). Together, these residues establish a hydrogen-bonding and hydrophobic network that recognizes the hydroxyl groups of D-glucose and stabilizes the occluded intermediate, a prerequisite for efficient transport (Holman, 2018; Mueckler et al., 1994; Villagran et al., 2023). The corresponding residues in yeast Hxt1—Q209 (TM5), N370 (TM8), and W473 (TM10)—fulfill analogous structural roles and are essential for glucose uptake, as evidenced by the near-complete loss of transport activity following their substitution (Roy et al., 2015).

**FIGURE 1:**
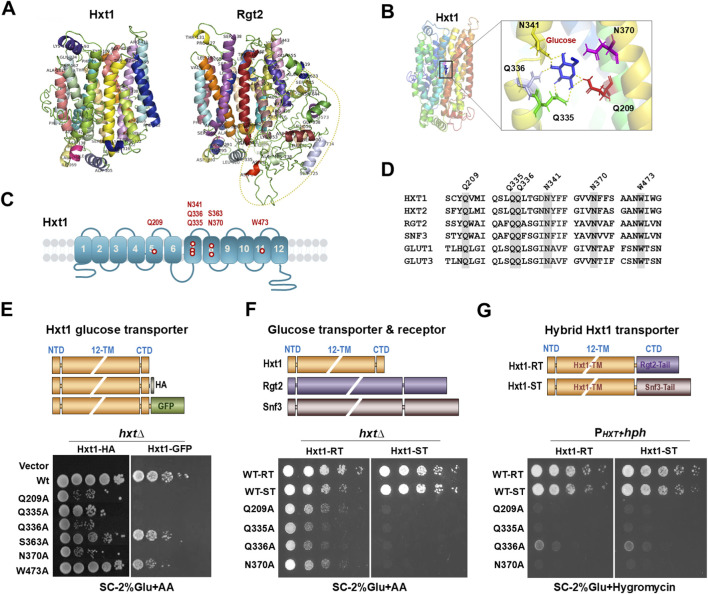
Hxt1 transport activity is restricted by its C-terminal cytoplasmic tail. **(A)** The glucose sensing receptors Rgt2 and Snf3 are distinguished from Hxt glucose transporters by their long C-terminal cytoplasmic tails (red ellipses), which likely confer sensing rather than transport function. Reproduced from Kim et al. (2023), with permission from Elsevier (CC-BY license, 6104860330842). **(B)** Homology model of Hxt1 generated with I-TASSER and visualized in PyMOL (Kim et al., 2023); glucose docking to the predicted binding site shown using AutoDock Vina (Trott and Olson, 2010). Reproduced from Kim et al. (2024). **(C)** Schematic model of Hxt1 transmembrane topology, with glucose-binding residues marked. **(D)** Clustal Omega was used for sequence alignment of yeast glucose transporters (Hxt1 and Hxt2), yeast GSRs (Rgt2 and Snf3), and human glucose transporters (GLUT1 and GLUT3). Adapted from Kim et al. (2024). **(E–G)** Schematic representation of the structures of Hxt1 and its variants Hxt1-HA, Hxt1-GFP, Hxt1-RT (Rgt2 C-terminal tail), and Hxt1-ST (Snf3 C-terminal tail). The *hxt*-null strain (*hxtΔ*) expressing the indicated genes were scored for growth on SC-medium containing 2% glucose with Antimycin A (AA, 1 mg/mL) **(E,F)**. The P_*HXT1*_ *-hph* reporter strains expressing the indicated *HXT1* wild-type and variants were scored for growth in a SC-2% glucose plate supplemented with 200 μg/mL hygromycin. Panels reproduced from Roy et al. (2015) **(E)** and Kim et al. (2024) **(F,G)**.

This one-to-one correspondence between GLUT1 and Hxt1 highlights the stringent structural requirements that govern substrate recognition and conformational coupling (Figures 1A–D). Q209 in Hxt1 likely mirrors the role of Q161 in GLUT1, forming hydrogen bonds with glucose hydroxyl groups during substrate entry and occlusion. N370, corresponding to N317 in GLUT1, is predicted to stabilize glucose within the binding pocket and may contribute to gating transitions that define the alternating-access mechanism (Kim et al., 2023). Likewise, W473, homologous to W412, provides a hydrophobic and aromatic interface that secures the glucose ring and helps maintain a transport-competent conformation (Kim et al., 2023; Roy et al., 2015).

Collectively, these conserved features reinforce the view that yeast and human glucose transporters operate through a shared mechanistic logic. Substrate binding within the central cavity triggers a series of salt-bridge and intrahelical rearrangements that drive the transition from the outward-to inward-facing state—a process that has remained fundamentally conserved across eukaryotic evolution.

## GFP attachment to Hxt1 can nonspecifically influence glucose binding

The use of protein tags, while often essential for experimental studies, can have unintended consequences on transporter function. In the case of Hxt1, the N-terminal domain contains lysine residues (K12 and K59) that are likely recognized by the E3 ligase Rsp5, targeting the transporter for endocytic internalization and vacuolar degradation under glucose-limited conditions—a regulatory mechanism that allows cells to adapt efficiently to nutrient fluctuations. By contrast, deletion of the C-terminal domain destabilizes Hxt1, resulting in rapid degradation and complicating functional analysis. We have therefore turned to C-terminal tagging as a strategy to probe transporter function while maintaining stability (Kim et al., 2024; Roy et al., 2015).

Not all tags, however, are functionally equivalent. The HA tag, comprising only nine amino acids and lacking any structured domain, is unlikely to perturb folding, conformation, or glucose-binding activity. Consequently, Hxt1-HA appears functionally comparable to untagged Hxt1. GFP, in contrast, is a large (∼27 kDa), folded protein that can interfere with protein conformation and flexibility. Consistent with this concern, GFP attachment to Hxt1 markedly reduces transport activity. Notably, the Q335A mutation completely abolishes transport, suggesting that the GFP fusion exacerbates the deleterious effects of mutations within the transmembrane domain (Figure 1E). This observation implies that large C-terminal tags can interfere with the conformational transitions required for the alternating-access transport mechanism, particularly when the transmembrane core is already destabilized (Roy et al., 2015).

Taken together, these findings serve as a cautionary example: while C-terminal fusions provide experimental convenience, their presence can unintentionally modulate transporter function, underscoring the importance of carefully considering tag size and structural impact when interpreting functional assays.

## Hxt1 variants carrying Rgt2 or Snf3 tails exhibit distinct glucose transport activities

The extended cytoplasmic C-terminal tails of the Rgt2 and Snf3 glucose-sensing receptors have long been recognized for their role in mediating glucose-dependent signaling rather than direct substrate transport (Figure 1A). Fusing these tails to Hxt1 have provided a compelling window into how regulatory domains can allosterically influence transporter function. Generation of Hxt1-RT (Rgt2 tail, 213 amino acids) and Hxt1-ST (Snf3 tail, 337 amino acids) demonstrates that, in the native Hxt1 transporter, these tail additions do not substantially alter Hxt1’s intrinsic glucose transport, reinforcing the idea that the tails primarily function in signaling (Kim et al., 2024).

However, the picture changes when key glucose-binding residues in Hxt1 are mutated. The impact of tail fusion becomes mutation-dependent: Rgt2-tail fusions can either enhance or reduce transport depending on the substituted residue, whereas Snf3-tail fusions consistently abolish transport across all tested mutants. Notably, the transport activities of RT-fused Hxt1Q209A or Hxt1N370A exceed those of the corresponding HA-fused variants, while RT-fused Hxt1Q335A exhibits lower activity than its HA-fused counterpart (Figure 1F). These observations suggest that the Rgt2 and Snf3 tails exert distinct allosteric effects on the transmembrane core, modulating the ability of the transporter to undergo conformational transitions required for glucose flux.

Taken together, these findings underscore a broader principle: under normal conditions, the YGSR tails decouple signaling from transport, effectively insulating glucose flux from regulatory activity. Yet, when the transporter core is perturbed, the tails reveal their influence on transporter conformation in a mutation-specific manner. This highlights the sophisticated interplay between regulatory domains and core transporter architecture, suggesting that even noncatalytic regions can meaningfully shape substrate transport when the system is stressed or destabilized.

## YGSR tails potentiate signaling while revealing decoupling from transport

Despite their distinct transport behaviors, Hxt1-RT and Hxt1-ST retain comparable glucose-signaling activity, emphasizing the modularity of transporter regulation. Transplanting the YGSR tails onto Hxt1 or Hxt2 converts them into glucose sensors capable of activating HXT gene expression. Interestingly, Rgt2 lacking its tail still signals when overexpressed, indicating that the tails are not strictly required for signal initiation but rather serve to stabilize or potentiate the signaling process.

Notably, the signaling capacity of Hxt1-RT and Hxt1-ST remains lower than that of native Rgt2 and Snf3 glucose receptors, likely reflecting the absence of glucose-dependent phosphorylation by Yck1/2, which normally stabilizes the glucose receptors in signaling-competent conformations (Kim et al., 2024). This effect is further amplified in Hxt1 mutants with substitutions at key glucose-binding residues, emphasizing that the structural integrity of the transporter core is essential for effective signal transduction. Interestingly, Hxt1-Q335A retains substantial transport when fused to short tags but loses both transport and signaling with longer tail fusions, whereas Hxt1-Q336A shows minimal transport regardless of tag yet retains partial signaling when fused to Rgt2 or Snf3 tails (Figure 1G). These observations suggest that specific residues function as critical communication nodes or molecular switches, enabling partial signal relay even when other interactions are compromised.

Overall, these findings illustrate that glucose transport and signaling are mechanistically coupled yet separable. YGSR tails primarily potentiate signaling, but effective transduction depends on the integrity of the transporter core. Subtle alterations in key residues can selectively disrupt signaling without abolishing transport, highlighting the finely tuned interplay between structural architecture and regulatory output in MFS transporters.

## Discussion

Our findings provide new insight into how the functional divergence between glucose transport and glucose sensing arises within the MFS transporter scaffold. Attachment of the receptor tails to Hxt1 has minimal impact on intrinsic transport, whereas deletion of the tail domain from Rgt2 (and possibly Snf3) fails to restore glucose transport. This indicates that the inability of these receptors to transport glucose stems from intrinsic features of their transmembrane cores rather than tail-mediated inhibition.

The effects of fusing glucose receptor tails become particularly informative in the context of Hxt1 mutants with alanine substitutions at key glucose-binding residues. These chimeras display distinct transport activities while largely losing signaling function. Specifically, Hxt1 variants fused to the Rgt2 tail retain low levels of transport, whereas fusion to the Snf3 tail nearly abolishes it, highlighting the differential modulatory roles of the two receptor tails. These observations suggest that tail identity can allosterically influence transporter conformation, with the Rgt2 tail permitting residual flexibility and the Snf3 tail imposing structural constraints on the transmembrane core.

The interplay between tail identity and specific glucose-binding residues further underscores a residue-specific mechanism of transport–signaling coupling. For example, Q335A maintains substantial transport with short tags but loses activity with longer tail fusions and cannot signal even when fused to glucose receptor tails, indicating a critical role in conformational transitions underlying both transport and signaling. In contrast, Q336A exhibits very low transport regardless of tail identity but retains partial signaling when fused to either the Rgt2 or Snf3 tail, suggesting this residue selectively mediates communication between transporter conformation and downstream signaling. Together, these results support a model in which adjacent residues in the glucose-binding site differentially contribute to transport and signal transduction, while YGSR tails modulate these processes in a residue-specific and context-dependent manner.

Beyond mechanistic insight, these findings carry broader implications for experimental design and transporter engineering. The widespread use of tagged proteins, particularly large C-terminal fusions like GFP, has been critical for localization, expression, and structural studies. However, our results underscore that such modifications can inadvertently distort transporter behavior—even when the transmembrane core remains unchanged. GFP fusions may alter membrane insertion, interfere with folding or oligomerization, or bias proteins toward non-physiological conformations. This is particularly relevant for structural studies, where tagged constructs may stabilize incomplete or artificially static states. These observations highlight the importance of using minimal or flexible tags, validating results with untagged controls, and carefully considering the regulatory effects of cytoplasmic domains in both functional and structural analyses.

Finally, these findings provide a conceptual framework for understanding the evolutionary split between glucose transporters and receptors. Rather than arising from wholesale divergence in transmembrane sequences, the functional separation may reflect adaptive interdomain constraints. Extended cytoplasmic tails appear to stabilize transmembrane conformations that are competent for glucose sensing but incompatible with active translocation, illustrating an elegant evolutionary strategy in which the structural scaffold of MFS proteins is repurposed to serve distinct physiological roles.

Overall, our analysis emphasizes the modular logic of transporter–receptor evolution: subtle alterations within the transmembrane core or cytoplasmic tails can selectively decouple signaling from transport, providing a versatile platform for functional diversification while preserving the fundamental MFS architecture.
